# Acute Onset Extrapyramidal Symptoms With Clozapine: A Report of a Rare Case

**DOI:** 10.7759/cureus.108683

**Published:** 2026-05-11

**Authors:** Shruthi Nandakumar, Santosh Ramdurg, Shivakumar Chaukimath, Manovijay B Kalasagond, Gouthami S G

**Affiliations:** 1 Department of Psychiatry, Shri B. M. Patil Medical College Hospital and Research Centre, Bijapur Lingayat District Educational Association (BLDE), Vijayapura, IND

**Keywords:** clozapine, electroconvulsive therapy, extrapyramidal symptoms, orofacial dyskinesia, tetrabenazine, treatment-resistant schizophrenia

## Abstract

Clozapine is considered the gold standard for the management of treatment-resistant schizophrenia (TRS) because of its superior efficacy and relatively low risk of extrapyramidal symptoms (EPS). However, rare cases of clozapine-associated EPS have been reported, challenging the widely held belief regarding its minimal propensity to induce movement disorders. We report the case of a 29-year-old female with TRS who developed acute-onset orofacial dyskinesia and marked psychomotor slowing within one week of initiating clozapine at a low dose of 25 mg/day. The patient exhibited involuntary movements involving the lips, tongue, and facial muscles, accompanied by slurred speech, difficulty with mastication and swallowing, and increased social withdrawal. Neurological examination revealed isolated orofacial dyskinesia without associated rigidity, tremor, or autonomic instability. Clozapine was discontinued promptly, and tetrabenazine was initiated, resulting in significant clinical improvement, with the Abnormal Involuntary Movement Scale (AIMS) score decreasing from 20 to 9. Owing to persistent psychotic symptoms and intolerance to clozapine, the patient was subsequently treated with electroconvulsive therapy (ECT). This case underscores that clozapine, despite its well-recognized favorable extrapyramidal safety profile, may rarely precipitate acute EPS even at low doses. Clinicians should remain vigilant during the initiation phase and monitor for atypical movement disorders to enable timely intervention and optimize outcomes in patients with TRS.

## Introduction

Schizophrenia is a chronic and severe psychiatric disorder characterized by disturbances in thought, perception, emotion, and behavior. Despite the availability of multiple antipsychotic medications, approximately 20-30% of patients do not achieve an adequate response to standard antipsychotic therapy, a condition referred to as treatment-resistant schizophrenia (TRS) [1]. Clozapine remains the gold standard treatment for TRS because of its superior efficacy in reducing persistent positive symptoms, lowering relapse rates, and decreasing the risk of suicide [2,3].

The unique receptor-binding profile of clozapine contributes to its distinctive clinical effects. Clozapine exhibits relatively weak antagonism at dopamine D₂ receptors along with strong antagonistic activity at serotonergic (5-HT2A/2C), muscarinic, histaminergic, and adrenergic receptors. This pharmacological profile is believed to underlie its low propensity to cause extrapyramidal symptoms (EPS) compared with first-generation antipsychotics and several other second-generation agents [4]. As a result, clozapine-induced EPS are generally considered uncommon and may be under-recognized in routine clinical practice.

Nevertheless, emerging evidence indicates that movement disorders such as dyskinesia, dystonia, and Parkinsonism can occur, although rarely, in patients treated with clozapine [5-7]. Reported risk factors for clozapine-associated extrapyramidal symptoms include prior exposure to dopamine-blocking antipsychotics, rapid dose escalation, individual susceptibility, and pharmacodynamic or pharmacogenomic variability. The mechanisms underlying these reactions remain incompletely understood but may involve individual susceptibility, prior exposure to dopamine-blocking agents, alterations in dopaminergic-serotonergic balance, and pharmacodynamic variability. In this report, we describe a rare case of acute-onset extrapyramidal symptoms following initiation of low-dose clozapine, highlighting the importance of careful monitoring during the early phases of treatment and awareness of atypical adverse reactions.

## Case presentation

A 29-year-old unmarried female from Karnataka, India, educated up to graduation and previously employed as a lecturer, presented with a two-year history of progressive psychiatric symptoms. Her premorbid personality was reported to be well adjusted, with adequate social, occupational, and interpersonal functioning prior to the onset of illness. The illness began insidiously approximately two years prior to presentation. Initially, she developed increased fearfulness, suspiciousness, and excessive concern about trivial matters. Over time, she became apprehensive about routine activities and gradually began to avoid stepping outside the home. Within a few weeks, she exhibited increasing social withdrawal, reduced interaction with family members, and heightened sensitivity to environmental stimuli, particularly loud noises.

As the illness progressed, she developed clear psychotic symptoms, including second-person auditory hallucinations and delusions of reference and persecution. Her behavior became increasingly disorganized, accompanied by a marked decline in self-care and occupational functioning, ultimately leading to resignation from her job. There was no history suggestive of mood episodes, substance use, seizures, or head injury.

The patient had received multiple trials of antipsychotic medications, including olanzapine, amisulpride, and quetiapine, each administered in adequate therapeutic doses and durations. Although partial improvement was noted initially, symptoms persisted and relapsed over time despite consistent medication adherence throughout the course of the illness. Given the inadequate response to pharmacotherapy, she underwent five sessions of electroconvulsive therapy, which resulted in moderate but transient improvement. Based on the failure of multiple antipsychotic trials, a diagnosis of treatment-resistant schizophrenia was established.

After baseline clinical and laboratory evaluation, including hematological and cardiac assessments, clozapine was initiated at a conservative dose of 25 mg/day in accordance with standard titration protocols for treatment-resistant schizophrenia. Prior to the initiation of clozapine, the patient had been maintained on quetiapine, which was gradually tapered and discontinued. There was no abrupt withdrawal of antipsychotic medication before clozapine initiation. The interval between discontinuation of the previous antipsychotic and initiation of clozapine was clinically uneventful, without abnormal involuntary movements. Electroconvulsive therapy had been completed several weeks prior to the onset of dyskinetic symptoms.

Within one week of initiating clozapine, the patient developed involuntary movements involving the lips, tongue, and facial muscles. These movements were continuous and distressing, resulting in slurred speech, difficulty in mastication and swallowing, and increased social withdrawal. She also demonstrated marked psychomotor retardation. Neurological examination revealed isolated orofacial dyskinesia without evidence of limb rigidity, tremor, abnormal reflexes, or cranial nerve involvement. There were no signs of autonomic instability, hyperthermia, or altered sensorium suggestive of neuroleptic malignant syndrome. 

**Figure 1:**
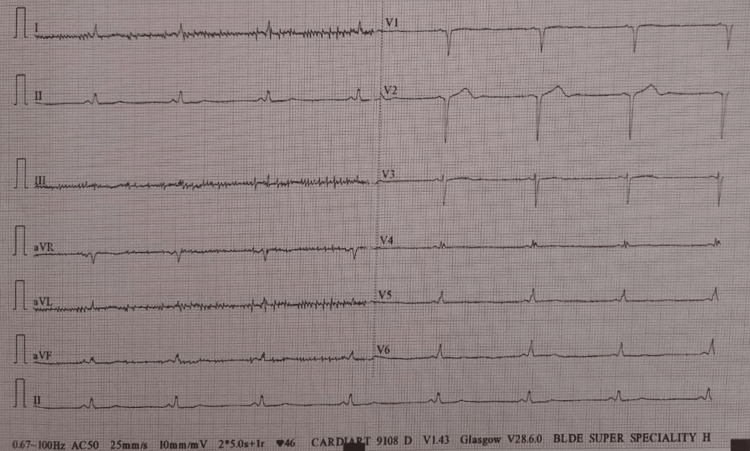
Electrocardiogram of the patient Electrocardiogram showing sinus bradycardia with a heart rate of 46 beats/minute. PR interval was 120 milliseconds and QT/QTc interval 480/420 milliseconds.

Routine laboratory investigations, including complete blood count, liver function tests, renal function tests, and serum electrolytes, were within normal limits, as summarized in Table 1. Electrocardiography revealed sinus bradycardia with a heart rate of 46 beats/minute, as shown in Figure 1. Similar findings were present on baseline ECG prior to clozapine initiation, suggesting that the bradycardia was pre-existing rather than drug-induced. The patient remained hemodynamically stable without cardiac symptoms, and no specific intervention was required apart from clinical observation.

**Table 1:** Laboratory Investigations g/dL: grams per deciliter; cells/mm³: cells per cubic millimeter; %: percentage; lakh/mm³: lakhs per cubic millimeter; fL: femtoliters; pg: picograms; mg/dL:  milligrams per deciliter; U/L: units per liter; SGPT: serum glutamic pyruvic transaminase; ALT: alanine aminotransferase; SGOT: serum glutamic-oxaloacetic transaminase; AST: aspartate aminotransferase.

Parameter	Result	Reference Range
Hemoglobin (Hb)	14.1 g/dL	12–16 g/dL
Total Leukocyte Count (TC)	5900 cells/mm³	4000–11000 cells/mm³
Neutrophils	59%	40–70%
Basophils	0%	0–1%
Eosinophils	2%	1–6%
Lymphocytes	35%	20–40%
Monocytes	4%	2–8%
Platelet Count	3.04 lakh/mm³	1.5–4.5 lakh/mm³
Mean Corpuscular Volume (MCV)	89.4 fL	80–100 fL
Mean Corpuscular Hemoglobin (MCH)	29.9 pg	27–33 pg
Mean Corpuscular Hemoglobin Concentration (MCHC)	33.6 g/dL	32–36 g/dL
Serum Creatinine	0.9 mg/dL	0.6–1.1 mg/dL
Total Bilirubin	0.4 mg/dL	0.2–1.2 mg/dL
Direct Bilirubin	0.2 mg/dL	0.0–0.3 mg/dL
Indirect Bilirubin	0.2 mg/dL	0.2–0.8 mg/dL
SGPT (ALT)	15 U/L	7–35 U/L
SGOT (AST)	20 U/L	10–40 U/L
Total Protein	6.8 g/dL	6.0–8.3 g/dL
Albumin	4.0 g/dL	3.5–5.0 g/dL
Globulin	2.8 g/dL	2.0–3.5 g/dL
A:G Ratio	1.4	1.0–2.0
Random Blood Sugar (RBS)	98 mg/dL	70–140 mg/dL

Additional investigations, including serum clozapine/norclozapine levels, thyroid function tests, serum calcium, magnesium, and creatine phosphokinase (CPK) levels, MRI brain, and evaluation for Wilson disease (serum ceruloplasmin, 24-hour urinary copper, slit-lamp examination), were not performed. These were limited by resource availability and low clinical suspicion, as the patient had no hepatic dysfunction, focal neurological deficits, cognitive fluctuation, or other features suggestive of Wilson disease, autoimmune encephalitis, or structural brain pathology. The close temporal association with clozapine initiation and improvement following drug discontinuation supported a probable drug-induced etiology.

Considering the temporal association with clozapine initiation, the medication was promptly discontinued, and tetrabenazine 25 mg/day was initiated on the same day that extrapyramidal symptoms were noted [8]. On reassessment approximately one week later, there was a marked reduction in involuntary movements, with the Abnormal Involuntary Movement Scale (AIMS) score improving from 20 to 9 [9]. Due to persistent psychotic symptoms and intolerance to clozapine, the patient was subsequently managed with electroconvulsive therapy as an alternative treatment strategy.

## Discussion

Clozapine occupies a unique position among antipsychotic medications because of its superior efficacy in treatment-resistant schizophrenia and its comparatively low propensity to produce extrapyramidal symptoms (EPS) [4]. This favorable neurological profile is largely attributed to its weak dopamine D₂ receptor antagonism combined with strong serotonergic (5-HT₂A) receptor blockade. Consequently, clozapine has traditionally been regarded as an antipsychotic with minimal risk of inducing movement disorders. However, sporadic reports of clozapine-associated EPS challenge this conventional understanding and suggest that such adverse effects, although rare, can occur in susceptible individuals. Compared to previously reported cases, the present case is notable for the very early onset of symptoms at a low dose (25 mg/day), which is rarely reported in the literature [5-7].

Several mechanisms have been proposed to explain the occurrence of EPS during clozapine therapy. One hypothesis involves complex interactions between serotonergic and dopaminergic neurotransmission within the basal ganglia. Clozapine’s modulation of serotonin receptors may indirectly influence dopaminergic pathways, potentially leading to dysregulation of motor control. Additionally, individual variability in dopamine receptor sensitivity or genetic susceptibility may contribute to the development of movement disorders. Prior exposure to dopamine-blocking antipsychotics may also play a role by inducing long-term alterations in basal ganglia circuits, thereby increasing vulnerability to EPS even when using antipsychotics with lower D₂ receptor affinity [4].

The present case is notable for the rapid onset of orofacial dyskinesia following initiation of clozapine at a very low dose of 25 mg/day. Such an early presentation is uncommon, as dyskinetic movements associated with antipsychotic therapy are typically reported after prolonged exposure [5-7]. Importantly, the patient had no documented abnormal involuntary movements during previous antipsychotic treatment, and there was no abrupt discontinuation of prior antipsychotic medication before clozapine initiation, making tardive dyskinesia and withdrawal-emergent dyskinesia less likely alternative explanations. The temporal association between clozapine initiation and symptom onset, along with the marked improvement following drug discontinuation, strongly suggests a probable causal relationship. Tetrabenazine, a vesicular monoamine transporter type 2 (VMAT2) inhibitor, reduces synaptic dopamine availability and is widely used in the management of hyperkinetic movement disorders, including drug-induced dyskinesias [8].

Early recognition of extrapyramidal symptoms is essential because continued exposure to the offending agent may exacerbate movement abnormalities and negatively affect treatment adherence. In the present case, discontinuation of clozapine, combined with pharmacological management with tetrabenazine, resulted in significant improvement in dyskinetic movements, as reflected by a reduction in the Abnormal Involuntary Movement Scale (AIMS) score [9]. Unlike tardive dyskinesia, which develops gradually and often persists despite drug withdrawal, the acute onset and partial reversibility observed in this patient indicate a probable drug-induced phenomenon. Causality assessment using the Naranjo Adverse Drug Reaction Probability Scale yielded a score of 7, indicating a probable association between clozapine and the observed extrapyramidal symptoms [10].

This case highlights the importance of careful monitoring for atypical neurological adverse effects during the early phases of clozapine treatment. Although rare, the possibility of clozapine-induced EPS should be considered when new movement abnormalities emerge shortly after treatment initiation. A limitation of this report is the absence of serum clozapine level monitoring and comprehensive neurological investigations to exclude all secondary causes of dyskinesia. However, the temporal relationship and improvement after clozapine withdrawal favored a probable adverse drug reaction. Greater awareness among clinicians may facilitate prompt recognition and timely intervention, thereby minimizing morbidity and optimizing treatment outcomes in patients with treatment-resistant schizophrenia.

## Conclusions

This case highlights a rare instance of acute extrapyramidal symptoms occurring shortly after initiation of low-dose clozapine in a patient with treatment-resistant schizophrenia. Although clozapine is widely regarded as having a low propensity to cause extrapyramidal side effects, clinicians should remain vigilant for atypical movement disorders, particularly during the early phase of treatment. Early recognition, prompt discontinuation of the offending agent, and appropriate symptomatic management are essential to minimize morbidity and ensure optimal patient outcomes.

## References

[1] Howes OD, McCutcheon R, Agid O (2017). Treatment-resistant schizophrenia: treatment response and resistance in psychosis (TRRIP) working group consensus guidelines on diagnosis and terminology. Am J Psychiatry.

[2] Kane J, Honigfeld G, Singer J, Meltzer H (1988). Clozapine for the treatment-resistant schizophrenic. A double-blind comparison with chlorpromazine. Arch Gen Psychiatry.

[3] Peitl V, Puljić A, Škrobo M, Nadalin S, Fumić Dunkić L, Karlović D (2023). Clozapine in treatment-resistant schizophrenia and its augmentation with electroconvulsive therapy in ultra-treatment-resistant schizophrenia. Biomedicines.

[4] Kapur S, Seeman P (2001). Does fast dissociation from the dopamine d(2) receptor explain the action of atypical antipsychotics?: a new hypothesis. Am J Psychiatry.

[5] Elliott ES, Marken PA, Ruehter VL (2000). Clozapine-associated extrapyramidal reaction. Ann Pharmacother.

[6] Das S, Purushothaman ST, Rajan V, Chatterjee SS, Kartha A (2017). Clozapine-induced tardive dyskinesia. Indian J Psychol Med.

[7] Sen M, Yesilkaya UH, Berkol DT (2022). A rare clinical finding associated with very low-dose clozapine: Pisa syndrome. Am J Ther.

[8] Jankovic J (1982). Treatment of hyperkinetic movement disorders with tetrabenazine: a double-blind crossover study. Ann Neurol.

[9] Guy W (1976). ECDEU Assessment Manual for Psychopharmacology. NIMH Psychopharmacology Research Branch,Division of Extramural Research Programs.

[10] Naranjo CA, Busto U, Sellers EM (1981). A method for estimating the probability of adverse drug reactions. Clin Pharmacol Ther.

